# Interim Effectiveness Estimates of 2023 Southern Hemisphere Influenza Vaccines in Preventing Influenza-Associated Hospitalizations — REVELAC–i Network, March–July 2023

**DOI:** 10.15585/mmwr.mm7237e1

**Published:** 2023-09-15

**Authors:** Ashley L. Fowlkes, Francisco Nogareda, Annette Regan, Sergio Loayza, Jose Mendez Mancio, Lindsey M. Duca, Paula Couto, Juliana Leite, Angel Rodriguez, Daniel Salas, Eduardo Azziz-Baumgartner, REVELAC–i Network, REVELAC–i Network, Estefania Benedetti, Andrea Pontoriero, Maria del Valle Juarez, Nathalia Katz, Maria Paz Rojas Mena, Carla Jimena Voto, Walquiria Aparecida Ferreira da Almeida, Daiana Araújo da Silva, Greice Madeleine Ikeda do Carmo, Francisco José de Paula Júnior, Miriam Teresinha Furlam Prando Livorati, Hellen Kássia Rezende Silva, Marcela Avendaño, María Fernanda Olivares Barraza, Patricia Bustos, Paula Rodríguez Ferrari, Natalia Vergara Mallegas, Rodrigo Fasce Pineda, Silvia Battaglia, Marta Von Horoch, Chavely Domínguez, Maria José Ortega, Elena Penayo, Cynthia Vázquez, Hector Chiparelli, Natalia Goñi, Karina Griot, Jose Eduardo Silvera, Daiana Tritten, Steven Tapia Villacís

**Affiliations:** ^1^Influenza Division, National Center for Immunization and Respiratory Diseases, CDC; ^2^Pan American Health Organization, Washington, DC; ^3^School of Nursing and Health Professions, University of San Francisco, San Francisco, California; ^4^Fielding School of Public Health, University of California Los Angeles, Los Angeles, California.; INEI-ANLIS–Dr. Carlos G. Malbrán, Buenos Aires, Argentina; INEI-ANLIS–Dr. Carlos G. Malbrán, Buenos Aires, Argentina; Ministry of Health, Buenos Aires, Argentina; Ministry of Health, Buenos Aires, Argentina; Ministry of Health, Buenos Aires, Argentina; Ministry of Health, Buenos Aires, Argentina; Ministry of Health, Brasília, Brazil; Ministry of Health, Brasília, Brazil; Ministry of Health, Brasília, Brazil; Ministry of Health, Brasília, Brazil; Ministry of Health, Brasília, Brazil; Ministry of Health, Brasília, Brazil; Ministry of Health, Santiago, Chile; Ministry of Health, Santiago, Chile; Ministry of Health, Santiago, Chile; Ministry of Health, Santiago, Chile; Ministry of Health, Santiago, Chile; Ministry of Health, Santiago, Chile; Ministry of Public Health and Social Welfare, Asunción, Paraguay; Ministry of Public Health and Social Welfare, Asunción, Paraguay; Ministry of Public Health and Social Welfare, Asunción, Paraguay; Ministry of Public Health and Social Welfare, Asunción, Paraguay; Ministry of Public Health and Social Welfare, Asunción, Paraguay; Ministry of Public Health and Social Welfare, Asunción, Paraguay; Ministry of Public Health, Montevideo, Uruguay; Ministry of Public Health, Montevideo, Uruguay; Ministry of Public Health, Montevideo, Uruguay; Ministry of Public Health, Montevideo, Uruguay; Ministry of Public Health, Montevideo, Uruguay; Ministry of Public Health, Montevideo, Uruguay

**Summary**

**What is already known about this topic?**

Effectiveness of seasonal influenza vaccine varies by season and circulating virus type.

**What is added by this report?**

The 2023 Southern Hemisphere seasonal influenza vaccine reduced the risk for influenza-associated hospitalizations by 52%. Circulating influenza viruses were genetically similar to those targeted by the 2023–24 Northern Hemisphere influenza vaccine formulation. This vaccine might offer similar protection if these viruses predominate during the coming Northern Hemisphere influenza season.

**What are the implications for public health practice?**

Vaccination remains one of the most effective ways to protect against influenza-associated complications. In anticipation of Northern Hemisphere influenza virus circulation, CDC recommends that health authorities encourage U.S. health care providers to administer seasonal influenza vaccine to all eligible persons aged ≥6 months.

## Abstract

Evaluations of vaccine effectiveness during the March–September Southern Hemisphere influenza season can provide valuable information for countries currently experiencing the influenza season and preceding the October–May Northern Hemisphere influenza season. Since 2013, multiple countries have participated in the Network for the Evaluation of Vaccine Effectiveness in Latin America and the Caribbean–influenza (la Red para la Evaluación de Vacunas en Latino América y el Caribe—influenza [REVELAC–i]) to estimate and monitor vaccine effectiveness (VE) in preventing severe acute respiratory infection (SARI)–associated hospitalization. Based on data contributed by Argentina, Brazil, Chile, Paraguay, and Uruguay on 2,780 SARI patients hospitalized during March 27–July 9, 2023, the adjusted VE against SARI hospitalization associated with any influenza virus during the 2023 Southern Hemisphere season was 51.9% (95% Confidence Interval [CI] 39.2%–62.0%), including 55.2% (95% CI: 41.8%–65.5%) against the predominating A(H1N1)pdm09. These early, interim estimates, provided before the expected end of seasonal influenza virus circulation, suggest that vaccination substantially reduced the risk for severe influenza illnesses, underscoring the benefits of influenza vaccination. In anticipation of Northern Hemisphere influenza virus circulation, the World Health Organization and CDC recommend that health authorities encourage health care providers to administer annual influenza vaccination to all eligible persons, particularly emphasizing the importance of vaccination for persons at increased risk for severe outcomes (e.g., very young children, persons with preexisting health conditions [including pregnant women], and older adults).

## Introduction

In Latin American and Caribbean countries, influenza viruses have been associated with 716,000–829,000 respiratory hospitalizations and 41,007–71,710 deaths each year (*1*). Despite a history of high influenza vaccination coverage in the region, declines in coverage since 2019 have been observed. Systematic monitoring of influenza and COVID-19 vaccine effectiveness (VE), as is conducted through the Pan American Health Organization’s Network for the Evaluation of Vaccine Effectiveness in Latin America and the Caribbean–influenza (la Red para la Evaluación de Vacunas en Latino América y el Caribe—influenza [REVELAC–i])^†^ can be helpful in supporting public messaging to improve vaccination coverage (*2*). In addition, VE estimates from countries in the Southern Hemisphere during the March–September influenza season can provide insight into the effectiveness of vaccines for use during the subsequent October–May Northern Hemisphere influenza season (*3*).

## Methods

VE against influenza-associated hospitalization was estimated using a test-negative case-control study design to compare the odds of vaccination between hospitalized patients with a positive influenza test result (test-positive patients [case-patients]) and influenza test-negative hospitalized control patients. Patients meeting criteria for severe acute respiratory infection (SARI), defined as acute respiratory infection with a history of fever or documented temperature of ≥100.4°F [≥38°C] and cough, with onset during the preceding 10 days resulting in hospitalization, were identified through sentinel SARI surveillance using a standardized protocol (*4*). Respiratory specimens were collected and tested for influenza virus type and subtype by reverse transcription–polymerase chain reaction (RT-PCR) in national reference laboratories.

During March 27–July 9, 2023, midseason data were available and were pooled from 486 sentinel hospitals, including 11 in Argentina, 455 in Brazil, eight in Chile, two in Paraguay, and 10 in Uruguay. Because influenza was circulating in the Southern Hemisphere earlier than usual and before the start of influenza vaccination campaigns, the VE evaluation began 2 weeks after each country's annual national influenza vaccination campaign.^§^ Participating countries used a Southern Hemisphere formulation of egg-based influenza vaccines; the egg-based trivalent formulation contained antigens from an A/Sydney/5/2021 (H1N1)pdm09-like virus, A/Darwin/9/2021 (H3N2)–like virus, and B/Austria/1359417/2021 (B/Victoria lineage)–like virus, with the egg-based quadrivalent formulation also containing B/Phuket/3073/2013 (B/Yamagata lineage)–like virus (*5*).

The evaluation population was restricted to SARI patients in three mutually exclusive target groups for vaccination based on national immunization policies. The criteria for each group varied slightly by country, but included 1) young children, 2) persons with preexisting conditions, and 3) older adults.^¶^ Case-patients were defined as SARI patients with a positive RT-PCR influenza test result. Control patients were defined as SARI patients with negative RT-PCR test results for both influenza and SARS-CoV-2. Patient vaccination status was ascertained via linkage to national electronic immunization records using unique patient identifiers.

Patients who received ≥1 dose of the 2023 season influenza vaccine ≥14 days before symptom onset were considered vaccinated; those who did not receive any influenza vaccine during the 2023 season before symptom onset were considered unvaccinated. Patients vaccinated <14 days before symptom onset or who had positive SARS-CoV-2 RT-PCR test results were excluded from the evaluation to avoid the risk for confounding (*6*).

VE was estimated using mixed effects logistic models adjusting for age in years (fit as cubic spline), week of symptom onset (fit as cubic spline), and presence of at least one preexisting condition, and accounting for country as a random effect. Analyses were stratified by influenza type and subtype (when available) and presented when data were sufficient (at least five patients contributing to all strata) or when the 95% CI of the VE estimate was <140%. P-values <0.05 and 95% CIs that did not include zero were considered statistically significant. This activity was reviewed by CDC and conducted consistent with applicable federal law and CDC policy.**

## Results

During March 27–July 9, 2023, a total of 3,974 SARI hospitalizations among persons prioritized to receive influenza vaccination in Argentina, Brazil, Chile, Paraguay, and Uruguay were identified. Among these, 1,194 were excluded due to a positive SARS-CoV-2 test result (405), influenza test result missing (278), vaccinated <14 days before symptom onset (163), receipt of the 2022 influenza vaccine <120 days before 2023 vaccine availability (164), respiratory specimen collected >10 days after symptom onset (82), missing vaccine or demographic information (83), or a duplicated report (19). After these exclusions, 2,780 SARI patients remained in the final analytic sample for the three vaccination target groups, including 1,262 (45.4%) young children, 388 (14.0%) persons with preexisting conditions, and 1,130 (40.6%) older adults (Table 1). Overall, 88 (3.2%) SARI patients were from Argentina, 918 (33.0%) from Brazil, 1,158 (41.7%) from Chile, 167 (6.0%) from Paraguay, and 449 (16.1%) from Uruguay. Influenza viruses were detected among 900 (32.4%) SARI patients, including 815 (90.6%) influenza type A and 85 (9.4%) type B viruses, with a higher percentage of specimens testing positive during earlier surveillance weeks than during the average 2011–2019 circulation (Figure). Among 673 (82.6%) of the 815 influenza A viruses that were subtyped, 668 (99.3%) were A(H1N1)pdm09 and five (0.07%) were A(H3N2); all 85 B lineages were B/Victoria. Influenza detections varied by target group: nearly one half (547; 48.4%) were detected in older adults with SARI, approximately one third (139; 35.8%) in persons with preexisting conditions, and 214 (17.0%) in young children (p-value <0.001). Overall, 23.9% of SARI patients were vaccinated. Although this proportion did not vary by target group, significant differences in vaccination prevalence were observed among SARI patients by country, ranging from 9.4% in Uruguay to 37.0% in Chile.

**TABLE 1:** Seasonal vaccination status and influenza test results and among hospitalized patients with severe acute respiratory illness, by selected characteristics — REVELAC–i Network, March–July 2023

Characteristic	SARI patients					
	Total, no.	Vaccinated* no. (row %)	p-value^†^	Influenza test result, no. (%)		p-value^†^
				Positive	Negative	
**Overall**	**2,780**	**664 (23.9)**	**—**	**900 (32.4)**	**1,880 (67.6)**	**—**
**Country**						
Argentina	88	24 (27.3)	<0.001	22 (25.0)	66 (75.0)	<0.001
Brazil	918	150 (16.3)		438 (47.7)	480 (52.3)	
Chile	1,158	428 (37.0)		301 (26.0)	857 (74.0)	
Paraguay	167	20 (12.0)		42 (25.1)	125 (74.9)	
Uruguay	449	42 (9.4)		97 (21.6)	352 (78.4)	
**Target group^§^**						
Young children	1,262	305 (24.2)	0.76	214 (17.0)	1,048 (83.0)	<0.001
Persons with preexisting conditions	388	87 (22.4)		139 (35.8)	249 (64.2)	
Older adults	1,130	272 (24.1)		547 (48.4)	583 (51.6)	
**Preexisting conditions** ^¶^						
One or more condition	1,624	374 (23.0)	0.21	677 (41.7)	947 (58.3)	<0.001
No preexisting condition	1,156	290 (25.1)		223 (19.3)	993 (80.7)	
**Sex**						
Female	1,480	347 (23.4)	0.56	512 (34.6)	968 (65.4)	0.01
Male	1,300	317 (24.4)		388 (29.9)	912 (70.1)	
**Influenza test result**						
Negative	1,880	526 (28.0)	—	—	1,880 (100)	—
Positive (all)	900	138 (15.3)		900 (100)	—	
Influenza A	815	128 (15.7)		815 (100)	—	
Influenza A/H1N1	668	102 (15.3)		668 (100)	—	
Influenza A/H3N2	5	0 (—)		5 (100)	—	
Influenza B	85	10 (11.8)		85 (100)	—	

**Abbreviations:** RT-PCR = reverse transcription–polymerase chain reaction; SARI = severe acute respiratory infection.

* Patients who received ≥1 dose of the 2023 season influenza vaccine ≥14 days before symptom onset were considered vaccinated; patients who did not receive any influenza vaccine during the 2023 season by the time of symptom onset were considered unvaccinated. Patients vaccinated 0–14 days before symptom onset or with positive SARS-CoV-2 RT-PCR test results were excluded from the evaluation to avoid the risk of confounding.

**^†^** A Pearson’s chi-square test was used to ascertain whether there were differences in the numbers of persons who were vaccinated and unvaccinated or who received positive and negative influenza test results.

^§^ Target groups are presented as mutually exclusive groups of persons considered at high risk for severe outcomes associated with influenza infection. Within countries, young children were defined as the following age groups: Argentina = 6 months–2 years; Paraguay = 6 months–3 years; Chile and Uruguay = 6 months–5 years; and Brazil = 6 months–6 years. Older adults were defined as aged ≥60 years (Brazil and Paraguay) and aged ≥65 years (Argentina, Chile, and Uruguay). The preexisting conditions tracked by REVELAC–i are asthma, cancer, hypertension, diabetes, cardiovascular disease, respiratory disease (excluding asthma), obesity, and immunocompromise.

^¶^ Number of all SARI patients with preexisting conditions, including young children and older adults.

**FIGURE:**
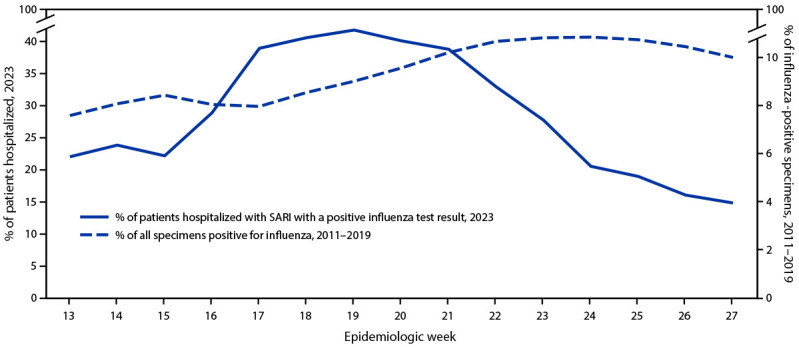
Percentage of patients hospitalized with severe acute respiratory infection with positive influenza virus test results,* by epidemiologic week, among 2011–2019 sentinel surveillance reports and hospitalized patients (N = 2,780) — REVELAC–i Network, Argentina, Brazil, Chile, Paraguay, and Uruguay, March–July 2023 **Abbreviation:** SARI = severe acute respiratory infection. * By reverse transcription–polymerase chain reaction testing at national reference laboratories.

Among SARI patients, 15.3% of case-patients had received a 2023 seasonal influenza vaccine, compared with 28.0% of control patients (Table 2). Overall adjusted VE against influenza-associated SARI hospitalization was 51.9%. VE among young children was 70.2%, and among older adults was 37.6% When limited to influenza A(H1N1)pdm09, which predominated during the 2023 Southern Hemisphere season, VE against SARI hospitalization was 55.2%, similar to overall estimates and consistent among target populations. Estimated VE for influenza B (Victoria) was not statistically significant (95% CI included zero) (Table 2), and insufficient numbers of vaccinated case-patients with influenza A(H3N2) precluded estimation of virus-specific VE.

**TABLE 2:** Interim 2023 southern hemisphere seasonal influenza vaccine effectiveness against all influenza types A and B and against virus type A(H1N1)pdm09 — REVELAC–i Network, March–July 2023

Influenza type/Target group^§^	Influenza test-positive case-patients*		Influenza test-negative control patients		Vaccine effectiveness^†^	
	Total	Vaccinated no. (%)	Total	Vaccinated no. (%)	Unadjusted % (95% CI)	Adjusted^†^ % (95% CI)
**Influenza A and B**						
Overall	900	138 (15.3)	1,880	526 (28.0)	53.3 (42.4 to 62.4)	51.9 (39.2 to 62.0)
Older adults	547	96 (17.6)	583	176 (30.2)	50.8 (34.1 to 63.3)	37.6 (13.1 to 55.2)
Children	214	19 (8.9)	1,048	286 (27.3)	74.0 (57.3 to 85.0)	70.2 (50.3 to 82.1)
Persons with preexisting conditions	139	23 (16.5)	249	64 (25.7)	42.7 (0.3 to 67.8)	38.0 (−10.8 to 65.3)
**Influenza A/H1N1**						
Overall	668	102 (15.3)	1,880	526 (28.0)	53.6 (41.2 to 63.6)	55.2 (41.8 to 65.5)
Older adults	422	70 (16.6)	583	176 (30.2)	54.0 (36.6 to 66.8)	42.7 (18.5 to 59.8)
Children	120	10 (8.3)	1,048	286 (27.3)	75.8 (52.9 to 88.9)	75.3 (52.1 to 87.3)
Persons with preexisting conditions	126	22 (17.5)	249	64 (25.7)	38.9 (−7.6 to 66.1)	43.0 (−6.7 to 69.5)
**Influenza B**						
Overall	85	10 (11.8)	1,880	526 (28.0)	65.7 (32.6 to 84.3)	46.2 (−7.9 to 73.2)

* Reverse transcription polymerase–chain reaction testing for influenza was conducted at national reference laboratories.

**^†^** Vaccine effectiveness was estimated using mixed effects logistic models adjusting for age in years (fit as cubic spline), week of onset of symptoms (fit as cubic spline), and presence of at least one preexisting condition and accounting for country as a random effect.

^§^ Within counties, young children were defined as the following age groups: Argentina = 6 months–2 years; Paraguay = 6 months–3 years; Chile and Uruguay = 6 months–5 years; and Brazil = 6 months–6 years. Older adults were defined as aged ≥60 years (Brazil and Paraguay) and aged ≥65 years (Argentina, Chile, and Uruguay).

As of August 15, 2023, a total of 1,031 influenza viruses had been genetically characterized by CDC, and the five countries’ laboratories reported the isolates to the Global Initiative on Sharing All Influenza Data.^††^ The majority of viruses were characterized as belonging to the same genetic clades as the 2023 Southern Hemisphere vaccine components: among 570 A(H1N1)pdm09 viruses, 309 (54.2%) belonged to subclade 5a.2a.1, 259 (45.4%) to subclade 5a.2a, and two (0.4%) to subclade 5a.1. Among six A(H3N2) viruses, three belonged to clade 3C.2a1b.2a.2 subclade 2a.1b and three to subclade 2b. All B/Victoria viruses belonged to subclade V1A.3a.2.

## Discussion

This interim evaluation of the 2023 Southern Hemisphere influenza vaccine formulation was conducted using data from five Southern Hemisphere countries and suggests that the current season’s trivalent and quadrivalent inactivated influenza vaccines are effective in reducing influenza-associated hospitalization. In particular, interim VE estimates indicate a significant reduction in hospitalization associated with the predominant influenza A(H1N1)pdm09 virus among young children and older adults. Vaccination is one of the most effective ways to prevent influenza and severe associated outcomes. Health authorities worldwide should encourage influenza vaccination for persons at increased risk for severe disease, including young children, persons with preexisting health conditions, and older adults, as well as those at increased risk for exposure to or transmission of influenza virus, such as health care personnel (*3*,*7*).

Despite the encouraging influenza VE, fewer than 30% of persons identified through REVELAC–i were vaccinated against influenza before their illness onset. The current findings were consistent with an interim report from Peru, where unadjusted VE against RT-PCR–confirmed influenza illness was 62%, but only one in five persons with medically attended respiratory illness had sought vaccination during 2023 (*8*), which is lower than the historical norm (*2*). Evaluations of influenza illness and vaccine knowledge, attitudes, and practices and deployment of World Health Organization (WHO) influenza post-introduction evaluations might help health authorities understand the reasons for reduced vaccination coverage since the COVID-19 pandemic and optimize influenza vaccination coverage in the future.^§§^

Although the timing and intensity of influenza epidemics in one hemisphere are not necessarily predictive of subsequent epidemics in the opposite hemisphere, this report might help health officials in Northern Hemisphere jurisdictions prepare for a potentially early influenza season and highlight the benefits of vaccination. In recent weeks, most influenza detections in the United States have identified A(H1N1)pdm09 and B/Victoria viruses (*9*). This is a similar pattern to that identified among the evaluated Southern Hemisphere countries, providing an encouraging outlook for vaccine protection from current influenza A(H1N1)pdm09 and influenza B/Victoria, because the Northern Hemisphere vaccine formulation contains antigenically similar A/Victoria/4897/2022 (H1N1)pdm09–like virus for egg-based vaccines and A/Wisconsin/67/2022 (H1N1)pdm09–like virus for cell-based vaccines. Nevertheless, whether influenza A(H1N1)pdm09 will remain the predominant virus in the United States during the October 2023–May 2024 Northern Hemisphere influenza season is unclear.

In advance of the WHO Vaccine Composition Meeting in September 2023, these data suggest that the influenza A/Sydney/5/2021 (H1N1)pdm09–like and B/Austria/1359417/2021 (B/Victoria lineage) viruses in the Southern Hemisphere vaccine formulation confer protection against influenza hospitalization; however, for the A/Darwin/9/2021 (H3N2)–like virus, VE estimates from other countries in the Southern Hemisphere, such as South Africa, which has a more substantial influenza A(H3N2) cocirculation, will be needed (*10*). Influenza B/Yamagata lineage has not circulated globally since 2020, and ongoing monitoring will be needed to determine if B/Yamagata antigens should remain in future influenza vaccine formulations.

### Limitations

The findings in this report are subject to at least five limitations. First, the VE estimates are preliminary and represent only a 3-month period with a smaller analytic sample, resulting in some VE estimates with wide CIs. Second, nearly 25% of the 1,194 otherwise eligible patients were missing RT-PCR results for influenza and were excluded from analysis. Although the REVELAC–i protocol indicates RT-PCR testing for all patients meeting SARI criteria, limited hospital resources for surveillance specimen collection during high-incidence periods might result in incomplete testing. Third, although statistical models accounted for important sources of confounding, the potential for unmeasured confounding associated with the likelihood of hospitalization or propensity for vaccination remains. Fourth, VE estimates from the Southern Hemisphere are important for supporting routine influenza vaccination programs, but these results might not be generalizable to other countries in the region with different vaccine target groups, timing of vaccination campaigns, or viral clade circulation. Finally, this analysis was unable to disaggregate previously unvaccinated children aged 6 months–9 years who received 2 doses of influenza vaccine from those receiving only 1 dose; thus, it is possible that influenza VE among young children restricted to those who received 2 doses of influenza vaccines would have been higher than VE among those with only a first, priming dose (*3*).

### Implications for Public Health Practice

Preliminary REVELAC–i data from five Southern Hemisphere countries suggest that influenza vaccines were effective in preventing more than one half of influenza-associated hospitalizations among young children, persons with preexisting conditions, and older adults. The 2023–24 Northern Hemisphere influenza vaccine formulation contains antigenically similar A/Victoria/4897/2022 (H1N1)pdm09–like virus (egg-based vaccines) and A/Wisconsin/67/2022 (H1N1)pdm09–like virus (cell-based vaccines) and might offer similar protection against influenza A(H1N1)pdm09 if these viruses predominate during the 2023–24 Northern Hemisphere influenza season. Evaluating influenza VE through integrated regional networks such as REVELAC–i provides valuable information that can guide public health practice. CDC and WHO recommend that health officials should encourage all eligible persons aged ≥6 months to seek influenza vaccination in accordance with national recommendations and to take other measures, including avoiding close contact with persons who are ill, to protect against influenza and potentially severe associated outcomes (*3*,*7*). Persons in the Northern Hemisphere who are at increased risk for influenza-associated complications, including children aged <2 years; persons with asthma; obesity; neurologic or neurodevelopmental conditions; blood disorders; chronic lung conditions; or endocrine, kidney, liver, metabolic, or heart diseases; who have had a stroke; or have a weakened immune system are especially encouraged to receive an influenza vaccination by September or October (*7*).

## Footnotes

* These authors contributed equally to this report.

†
https://www.paho.org/en/network-evaluation-vaccine-effectiveness-latin-america-and-caribbean-influenza-revelac-i

§ Argentina: March 17; Brazil’s Amazonas and Para states: March 11; other Brazil states: April 10; Chile: March 15; Paraguay: April 10; and Uruguay: April 18.

¶ Young children were defined as the following age groups: Argentina = 6 months–2 years; Paraguay = 6 months–3 years; Chile and Uruguay = 6 months–5 years; and Brazil = 6 months–6 years. Older adults were defined as aged ≥ 60 years (Brazil and Paraguay) and aged ≥65 years (Argentina, Chile, and Uruguay). The preexisting conditions tracked by REVELAC–i are asthma, cancer, hypertension, diabetes, cardiovascular disease, respiratory disease (excluding asthma), obesity, and immunocompromise.

** 45 C.F.R. part 46.102(l)(2), 21 C.F.R. part 56; 42 U.S.C. Sect. 241(d); 5 U.S.C. Sect. 552a; 44 U.S.C. Sect. 3501 et seq.

††
https://gisaid.org/

§§
https://apps.who.int/iris/handle/10665/70436
